# Physical literacy, health and interactive aging: a position paper

**DOI:** 10.3389/fspor.2024.1346802

**Published:** 2024-03-27

**Authors:** Rebecca J. Lloyd, Stephen Smith, Derya Sahingil

**Affiliations:** ^1^ Interdisciplinary Function2Flow Research Unit, Faculty of Education, University of Ottawa, Ottawa, ON, Canada; ^2^ Interdisciplinary Function2Flow Research Unit, Faculty of Education, Simon Fraser University, Burnaby, BC, Canada

**Keywords:** physical literacy, active aging, older adult, joy, interactive flow

## Abstract

Physical literacy (PL), a concept commonly associated with the early years, physical education, and youth sport development, can become a meaningful determinant of health and longevity for the adult and older adult population. A review of 55 recent publications from 2018 to 2023 that encompassed physical literacy conceptual frameworks, assessments, and intervention-based studies was undertaken through an heuristic inspired by the philosophy which gave birth to PL. With particular interest in how PL has evolved in response to the needs of an aging population, this position paper tracks a key shift in focus from the individual to the relational context. It references positive interaction and social participation in recent models as significant features of an across-the-lifespan PL perspective.The concluding position is that fostering joyful inter-action be at the heart of PL promotion, resource development and assessment practices, especially in the case of an aging population.

## Introduction

Physical literacy (PL) is a widely accepted concept that aligns physical activity with cognitive, affective, and behavioural attributions (1–6). It has been the focus of a large number of recent narrative, scoping and systematic reviews [e.g., (1, 7–15)]. Some of the recurring criticisms within these reviews have to do with the overemphasis on tying PL to the early and school-age years [e.g., (7); Edwards et al. 2018], associating PL narrowly with acquiring a select number of fundamental “sport” skills [e.g., (6, 16–18)], and the challenge in assessing an attitude (19, 20) by either breaking it into distinct parts or operationalizing it as an end-state PL determinant (1, 21, 22). While such concerns about PL conceptualizations prevail, we are inspired by the co-existing literacies perspective offered by Young et al. (18) in that different ways of conceiving of and assessing PL may help to collectively promote physical activity and health throughout the lifecourse.

## An interpretive approach

In presenting this position paper, the historic roots as well as the current definition of PL will be introduced to provide context for understanding how PL has been adapted and applied to the adult and older adult populations. The approach we take is inspired methodologically by Greenhalgh et al. (23) and Boell and Cecez-Kecmanovic (24) who suggest that a narrative, interpretive approach may be viewed as complementary, rather than inferior to, systematic reviews. This approach is premised on PL promotion from the outset where proponents drew upon phenomenological considerations of motility and meaning-making in advancing “the position that a physically literate individual should be able to articulate basic aspects of his/her embodiment” [(25), p. 288], which is to say, of each person's “unique motile potential” (p. 291). Our aim in this narrative interpretation is thus not just to expand but to deepen PL understandings particularly as they apply to older adults.

We aspire, phenomenologically and interpretively speaking, to a strong, oriented, rich and deep reading [cf. (26, 27)] of the pertinent PL literature. In this regard for the importance of PL, our approach is not so much biased towards a certain conception of PL as it is mindful of bringing one's own and others' life experiences to the fore in order to explicate more fully the circumstances in which viable models and practices of PL make good sense. This phenomenologically-informed, experientially-sourced, interpretive approach rests upon well-accepted precepts of the positioned address of phenomena of deep and abiding interest [cf. (28, 29)] and is in keeping with the task of interrogating essentially positioned “intuitions” and “insights” that may then be posited as foundational to scientific investigation (30). With a background in phenomenology and physical education [i.e., (31–46), Lloyd and Smith 2022, 2006] and a recent research interest in active aging (38, 47), we bring such an experientially-oriented, interpretive framework to bear upon how PL has been conceptualized, adapted and applied to adults and older populations. Our approach is intended to support an evolving sense of PL arising out of the good sense made of the concept so far.

In addition to reviewing foundational texts and studies in the field [i.e. (2–6, 19, 25, 48–54)], we searched for new publications from the last five years that were indexed in the following databases: ERIC (ProQuest), Scopus, MEDLINE (Ovid),and SPORTDiscus (EBSCOhost). For each of these databases the keywords we used were: “physical literac*” AND (aging OR senior* OR “older adult*” OR elder* OR “active for life”) AND (social OR relation* OR community OR isolation OR lonely*). In addition, we searched Google Scholar using the following search terms: (“physical literacy” OR “physical literacies”) AND (aging OR seniors OR “older adults” OR elderly OR elders OR “active for life”) AND (social OR relations OR community OR isolation OR loneliness). Over 295 publications were retrieved, however we limited our contextual readings to 55 based on their relevancy to our focus on PL, active aging, and health. These publications included:

• Five studies focused on the definitions and perspectives of PL (16, 55–58),
• Six systematic reviews (7, 9, 15, 21, 59, 60),
• Five scoping reviews (1, 10, 11, 13, 61),
• One integrated review (12),
• A narrative review (14),
• Twenty-six studies of healthy aging (7, 8, 10, 14, 15, 18, 22, 62–80),
• Four articles addressing PL measurement and measuring tools [(9, 13, 21, 61); Robinson and Randall, 2017],
• Five articles on perceived PL instruments and questionnaires (81–85), and
• Two articles that posed new PL models for active aging (12, 86).

## Evolving conceptions of physical literacy

It is worth revisiting when and why PL was first introduced in order to better understand how the concept has been adapted to include the adult and older adult population. Inspired by the holistic perspective of monism, and intention to foster a “literacy of the motile aspects of the human embodied dimension” [(53), p. 4] that would make a significant contribution to the quality of one's life, United Kingdom physical education scholar and founder of the International Physical Literacy Association (IPLA), Margaret Whitehead, coined the term “physical literacy.” It was intended to disrupt the dominance of Cartesian dualism in the separation of thought and action by emphasizing the moving body in physical activity research and physical education practice as a kinetically, kinesthetically and affectively sense-making entity (6, 25, 52–54). This “embodied” concept of physical literacy has subsequently become central to physical education reform (6, 87) as well as inspiring conceptual frameworks that now guide sport, recreation and coaching organizations such as Sport for Life in Canada, the Society of Health and Physical Educators (SHAPE) in the United States, Sport Australia, and Sport England.

While several definitions and iterations of PL have evolved over the years, a consensus was reached in 2015 among Canadian PL researchers, which has been adopted worldwide for the most part (49), to use the definition proposed by Whitehead and the IPLA. It states: “As appropriate to each individual, physical literacy can be described as the motivation, confidence, physical competence, knowledge, and understanding to value and take responsibility for engaging in physical activities for life” [(6), p. 8]. Accompanying this definition in Whitehead's (6, 52) PL texts, although not so commonly referenced, is a range of characteristics that mobilize PL into practical action. They can be summarized as the motivation, confidence, poise, thoughtful and sensitive perception, ability to work well independently and with others, ability to analyze effectiveness of movement performance, the understanding of holistic health principles, along with the self-assurance and self-esteem to take responsibility for choosing physical activity for life [(6), p. 12].

## Physical literacy and the older adult

Only 12% of older adults meet the physical activity guidelines set by the World Health Organization (1), which diminishes when factors of social isolation, being a visible minority, and low income are considered [(80), p. 335]. As Ciaccioni et al. (88) point out, “in the majority of aging people [this lack of physical activity] exacerbates the age-related decline of cardiorespiratory fitness, neuromuscular function, interlimb coordination, flexibility, endurance, and strength levels” (p. 2); and associated with such a significant decline in physical activity and subsequent loss of functional fitness is a decline in mental health and perceived “quality of life” (p. 11). The challenges to promoting PL across the lifespan are then all the greater when ablest and ageist assumptions of PL become increasingly apparent. Ablest assumptions tend to be imbedded not so much in the definition of PL we have cited, but in how this definition is operationalized as, for instance, by focusing on “physical competence” and designating the fundamental movements skills (FMS) of running, throwing and catching, kicking and trapping in normatively developmental patterns as precursors to participation in a variety of games and sports and activities throughout one's life (6, 16–18). Yet even more than an emphasis on learning to walk, run, roll, throw, catch, kick, etc. for the first time, “the older adult living with age-related physiological changes, […] may be more focused on retention rather than regaining past skills or learning new ones” [(86), p. 8]. Additionally, the daily movement patterns for this older adult may be quite unlike the generic techniques featured in FMS resources for physical education and youth sport which depict typically a “privileged and ableist focus on an individual's physical prowess [which is taken up as PL] by sport-focused organizations often to the exclusion of health, healthy living, community and social development, or inclusivity (disability) focused organizations” [(1), p.16]. One tends to forget that “the spectrum of human variation” [(89), p. 1568] is wider than “physical competence” indices of FMS acquisition.

Ageist assumptions similarly tend to go with these “physical competence” indices inasmuch as “motivation” and “confidence” are so closely tied to what is valued socially as demonstrable physical prowess. Promoting “youthfulness” at the expense of “a more positive image of older adults” [(90), p. 57], based on what these adults are motivated and confident to do, runs against sustaining PL into later life. Yet the inclinations to engage in enjoyable physical activity singly and with others, along with discernment as to what activities are good for one's health and social-emotional wellbeing, need not weaken at all with age. Furthermore, when the older adult takes center stage, emphasis need not be placed on the “individual” acquiring the motivation, knowledge and understanding to be active for life. A more interactive, communal, and ecological approach to the conceptualization and promotion of PL can be advanced [e.g., (57, 86)] to provide a broader reach and include not only peers and meaningful connections within the community (80) but also medical and rehabilitation professionals who have, for the most part, been excluded from PL research, resource development, and physical activity promoting policy (12).

In prioritizing the older adult, we gather literature support for considering how PL can incorporate multi-dimensional wellness, sustain the motivation to remain physically literate for later life, while suggesting also how PL and Active Aging can better inform the scope and direction of PL promotion in the earlier years. More than a concept for K-12 physical education and the development of young athletes, and having key relevance for the holistic promotion of ongoingly active lifestyles, PL can be advanced as a health-promoting praxis right across the lifespan (22).

## Physically literate for life

Despite the fact that Whitehead introduced PL as a “cradle to grave” phenomenon [(52), p. 18], most of the research, professional uptake and resources aimed at promoting PL has focused on the early and school aged-years [e.g., (9, 22, 91)]. To be physically literate for life means that we find ways to be active well beyond the school-aged years, when leaving home, starting a career, caring for family/loved ones, and experiencing mental, physical, and emotional challenges and setbacks as we age. In other words, if we are to take PL to heart, we will live life with a certain healthily active “disposition” (4, 6, 20, 58), continuing to find ways to prioritize and participate in physical activity no matter what obstacles get in our way.

What drives such a disposition, according to the late Len Almond, is a “love of being physically active” [(48), p. 123], although considering the “low levels of physical activity in the adult population, particularly those beyond the age of 65,” it seems that “we have failed to generate this love” (p. 129). Yet this activity disposition can be cultivated or rekindled at any age. Whether eight or eighty, engaging in purposeful physical activities “enhance lives and improve the quality of living” engenders this “love of being physically active” (p. 124).

More than 13 years have now passed since Almond (48) argued for the importance of cultivating a love of movement. If we are to radically shift the declining rates of physical activity in the adult and older adult populations (80), it seems we would be advised to again emphasize this love of movement. In doing so, we mean not merely the fun and satisfaction of moving competently and with confidence, but the joy and aliveness inherent to moving functionally, in good form, and with kinesthetic feeling and energetic flow (92–94). Embracing such a love of moment in a conceptually defensible way means, however, finding its validation in the ongoing articulation of PL.

In fact, the importance of positive affect is introduced in the conceptual model presented by Cairney et al. (49) which links PL to social and mental health. Rather than emphasizing the development of motor skills in isolation, as is the case in many of the PL studies of practitioner perceptions and resources, Cairney et al. (49) “argue that execution of motor performance on its own is insufficient for learning if it is not experientially linked with positive emotional states (*enjoyment*), which leads to a desire to repeat the skill and use it to engage in other activities such as sport (motivation), all within a particular *social* context or physical environment” (p. 373, *emphasis added*). While they conclude that these “motor, affect, social and cognitive components” [(49), p. 373] converge in multidimensional, interactive ways, we propose that more clarification as well as guidance is required if “enjoyment” and “social context” are to be prioritized in future PL research and practice. Furthermore, we can concur with Phoenix and Orr (95) “that within a policy context dominated by health outcomes, pleasure has remained a forgotten dimension of physical activity in older age” (p. 101). PL promotion for active aging aims to correct this omission.

Educators, coaches, peers as well as rehabilitation and medical professionals can certainly attune to the positively-felt sensations of movement, otherwise known as the “vitality affects” of movement expression, as defined by Stern (96), and elaborated on by Sheets-Johnstone (97–100). Quantitatively posed questions directed toward frequency and intensity of physical activity may then shift toward asking what kinds of activities bring kinesthetically-felt and synergistically-experienced joyfulness. And in keeping with the lived experiences of these physical activities, one can better ask: “What does joy actually feel like?” For instance: “Does the movement feel light, relaxed, and expansive vs. being heavy, tight and restricted? Do one's motions carry within them a resonance with the motions of others? Are we energetically and synergistically moving in concert with one another? Can it be said that we are expressing a shared joy?”

When joy wells up, as phenomenologist Chrétien (101) intimates, “everything expands. Our breathing becomes more ample, and our body suddenly stretches out of its self-confined corner and quivers with mobility” (p. 1). We may experience this spacious joy, moment to moment, one motion melding into the next, in cumulatively crescendoing ways. There are surges, rushes, and gushes of motional arousal that we can experience from cradle to grave and that constitute the primary vectors of movement joy (102). Images of elderly bodies going dutifully through prescribed exercises are countered by more positively active and interactive perspectives on active aging (50). These engaged bodies bring up life in one another. They find the spacious joy of “gesturally reciprocated motions” effected and affected in “kinesthetic conviviality” [(103), p. 157].

Eighty-four-year-old dancer, choreographer and motivational writer, Tharp (104), situates this joyously motile vitality, what she describes as “moving through life with energy and vigor” [(104), p. 4.], in ways of inviting older adults to take up more space. From walking, to greeting others to even spreading out one's items when we sit at a desk, she suggests striding, reaching, and more robust ways of interacting with others and the world, all of which expand the spaces and times of interactive agency and cultivated the love of movement.

Instantiating such expansive moments in specific kinetic functions and movement forms (otherwise restricted to fundamental movement skills) helps us appreciate not just the competencies that can be maintained but also the cultivation of movement confidence in the older population (12, 67, 82). By posing movement confidence as a part and parcel of motile expression, it becomes more than a psychological construct that may be assessed on a perceived scale, as is typically done in physical activity assessments [e.g., (9, 81)]. The amplitude, force, and tempo of moving with confidence singly and with others can be encouraged with older adults. Promoting PL can thus be understood as the requirement for PA leaders and facilitators to empathetically and e/motionally help older adults not only restore and sustain the range of their functional movement repertoires but also feel within the movements of which they are capable interpersonal and community connections. In doing so, moving joyfully remains within their reach and PL is the very means of doing so.

## Physical literacy, social participation and interactive flow

The welcome addition of social participation in Cairney et al.'s (49) conceptual model linking PL to health offers this very dimension to PL for which we now provide added perspective. In addition to the noteworthy interplay between “the environment (physical/social) and the nature of the task itself” Cairney et al. (49), as well as mentions of working well with others, in keeping with Whitehead's attributes of PL in “both cooperative and competitive situations” [(6), p. 40], we will elaborate further on the significance that moving with others has for the older adult population. We also provide further elaboration on how social participation connects to positive affect in what Cairney et al. (49) describe as the “multidimensional, experiential convergence of motor, affect, social and cognitive components” of PL [(49), p. 373].

An ecological model of PL developed for older adults by Jones et al. (86) emphasizes the integral connections between the individual, interpersonal, organizational, community and policy dimensions of PA promotion, planning and experience. While the individual dimension echoes the elements of PL in terms of motivation and confidence, physical competence, knowledge and understandings, and engagement in physical activities for life, the interpersonal dimension stresses the importance of support offered by friends, family, and health care providers. An additional model reframing PL for the aging adult was also put forward by Petrusevski et al. (12) and expands upon the role that rehabilitation professionals have in successful aging.

More than sources of support, however, merely experiencing movement with others, such as peers or other members of a group fitness or community class, carries signs of “improved well-being, improved social functioning, enhanced ability to carry out physical and emotional roles, and increased vitality” [(86), p. 11]. Similarly, Zimmer et al. (80) focused on the positive affects of social PA participation which counters the predominant narrative that “most physical activity research examining interpersonal processes has concentrated on social support for encouraging participation among older adults, and its association with psychosocial outcomes such as loneliness and social isolation” (p. 335).

When we combine the PL dimensions of positive affects (i.e., joyful action) with social participation, the theory of interactive flow [e.g., (93, 94, 105); Lloyd and Smith, 2006] can then offer a more suitable motivational construct than the heavily-cited self-determination theory of motivation [i.e., (6, 49)]. This former theory situates the positively-felt sensations and e/motions of flow within movement itself. Additionally, when social participation and interaction extend beyond the physical environment to include the spiritual dimensions of PL, we can be further inspired by recent studies that take into account eastern perspectives of PL where the motivational theory of flow is referenced by Sum and Whitehead (58) as well as Li et al. (55). Such studies equate what Csikszentmihalyi (106, 107) described as effortless action, in activities that are intrinsically satisfying and where pleasure and joy emerge from doing them for their own sake, with the Taoist concept of *wu wei*. To experience such pleasure in movement, *wu wei* is the perfect integration or balance between heaven (yang) and earth (yin) (58).

We encourage the uptake of flow theory for future PL research and approaches to assessment with the caveat that, rather than sequestering flow to an elitist “zone” of high performance, we recommend educative and progressive ways in which kinaesthetic feelings and synergistic micro-flows can be cultivated [i.e. (93, 94, 105),] no matter what the level of competency.

## A practical example of physical literacy

We take a moment to showcase an instance of PL that draws attention to how it may be experienced in the moment-to-moment, qualitative features of joyful, social interaction between an older adult and a fitness professional. Here we seek to ground PL even further in lived experiences which, as has been intimated from the outset of this paper, are the basis of phenomenological verification of the concept. Please note that “Ben” is a pseudonym for a personal training client who agreed to participate in the doctoral research study conducted by the first author of the present PL review (108).

…We approach another crosswalk. Ben just looks for a moment and then darts out before me. He likes to run out on the road. There are times when I have reached out and grabbed the back of his shirt in his moments of glee, watching the approaching traffic for fear we might not reach the other side in time. Ben even plays games with pedestrians. He likes to be the fastest person on the sidewalk. If there is another walker or jogger nearby, his focus is fixated ahead, pushing forward until we pass.

The sound of Ben's jog is a syncopated thud. The right foot often lands flat-footed, and the left quickly passes through to complete the cycle. It changes a little when I point out the sound or cue ease in the hips, but it is something that requires specific thought to refine–until we get to the light on Broadway. There is something different between that traffic light and the quieter street crossings. It gives so little time to cross safely to the other side.

Ben asks me to run ahead and get the light ready, so we don't have to stop. I run up, press the button, and it changes immediately. Ben, who is 10 steps behind, seizes the moment and overtakes me in a joyous stride. I feel the presence of a young, eager boy, speeding by my side. He floats across. The only sound is the swoosh in the air as he zooms by. I look up and see both of his arms swinging and he continues to run until the loud breaths catch him. The strident exhales return us to our walk and his heart rate starts to slow down.

Ben's sprint transcends time, his physical presence, the grounding reality of a heavy right foot, and the dysfunction of a right arm. His youthful energy caught him in a glide across the street to the other side and beyond. I push to catch up with him, laughing in the moment [.…] in a motion that transcended [old age]. [(108), 175–176]

Ben was seemingly heeding the advice of Almond (48) that we take seriously, as well as playfully, the importance of nurturing a love of movement. He and his personal trainer were demonstrating PL, in keeping with its definition which we cite again: “[a]s appropriate to each individual” and “as the motivation, confidence, physical competence, knowledge, and understanding *to value and take responsibility* for engaging in physical activities for life” (6), p. 8, *emphasis added*). It is the love of movement and its joyful expression, however, that we propose as being at the heart of valuing and taking responsibility for lifetime physical activity engagement. Fostering this love for ourselves and those with whom we work and play inspires us to consider how joyful action and inter-action can become foundational to PL promotion, practice and assessment. In this way, the world might just become a more joyous place for both old and young.

## Concluding position

Throughout this position paper, we have tried to show how positive affect and social participation are central to PL research. More than psychological and environmental constructs (49), there is a conceptual connection between this affectivity and relational sensibility and the phenomenological philosophy upon which PL is founded (6, 52). We press the case for adhering to the now widely accepted definition of PL while staying close to its lived experience data sources amidst the generalizing tendences of PL model designs and program and assessment frameworks. We make this case by drawing attention to the kinesthetic sensations and synergistic registers of moving with increasing confidence and competence while valuing the relational contexts where one can express freely with others a love of movement. In so doing, we trust that joyful, interactionally-vibrant and socially-engaged movement will increasingly come to undergird the relational dimension to PL which is emphasized in the models put forth by Jones et al. (86) and Petrusevski et al. (12) for older adults. We hope this focus on joy and relational connection inspires other PL researchers to also approach the teaching and promotion of active living, not only in the aging population, but across the entire lifecourse in this essentially interactional, health-sustaining manner.
